# Stability of Biologically Active Human Interferon α-2b in Transgenic “Long-Shelf Life” Tomatoes and Their Progeny

**DOI:** 10.3390/plants15182849

**Published:** 2026-09-17

**Authors:** Volodymyr Rudas, Olga Ovcharenko, Olha Yaroshko, Nadiia Zholobak, Vladyslav Moseiko, Nataliia Shcherbak, Bogdan Morgun, Taras Pasternak, Mykola Kuchuk

**Affiliations:** 1 Institute of Cell Biology and Genetic Engineering, National Academy of Sciences of Ukraine, Academika Zabolotnogo Street, 148-A, 03143 Kyiv, Ukraine; 90tigeryaroshko90@gmail.com (O.Y.); shcherbaknatalka@gmail.com (N.S.); bmorgun@icbge.org.ua (B.M.); nkuchuk@icbge.org.ua (M.K.); 2 Zabolotny Institute of Microbiology and Virology, National Academy of Sciences of Ukraine, Academika Zabolotnogo Street, 154, 03680 Kyiv, Ukraine; n.zholobak2018@gmail.com; 3 ESC “Institute of Biology and Medicine”, Taras Shevchenko National University of Kyiv, 64/13 Volodymyrska Street, 01601 Kyiv, Ukraine; moseykovlad@gmail.com; 4 Plantae Consulting ES, 03202 Elche, Spain

**Keywords:** human interferon α-2b, molecular farming, plant-based recombinant protein production, tomato, long-term heterologous protein storage in transgenic fruits, antiviral activity

## Abstract

Human interferon α-2b (HuIFNα-2b) is a cytokine with antiviral and antiproliferative activities and is widely used as a therapeutic protein. Here, we investigated the production of biologically active HuIFNα-2b in transgenic tomato plants. Long-shelf-life *Solanum lycopersicum* L. cv. ‘Shedevr 1’ plants were transformed via an *Agrobacterium*-mediated protocol using the binary vector pCB124 carrying *nptII* and *HuIFNα-2b* genes. PCR and RT-PCR analyses confirmed transgenes presence and expression in regenerated kanamycin-resistant plants. Antiviral activity was evaluated in vitro by the inhibition of vesicular stomatitis virus replication in MA-104 epithelial cells. Interferon activity reached 14.90 × 10^3^ IU g^−1^ FW in fresh fruit pulp and 854.00 × 10^3^ IU g^−1^ FW in leaves. Importantly, transgenic fruits retained substantial activity (2.42 × 10^3^–6.72 × 10^3^ IU g^−1^ FW) after three months of storage, with detectable activity (0.36 × 10^3^ IU g^−1^ FW) remaining after five months. Transgene inheritance and interferon activity were confirmed in the T_1_ and T_2_ generations. These results demonstrate the potential of long-shelf-life transgenic tomatoes as a platform for the production and extended storage of biologically active recombinant proteins, potentially reducing dependence on costly purification and cold storage procedures.

## 1. Introduction

In recent decades, plant-based platforms have been successfully applied for the efficient production of pharmaceutical proteins [1]. The advantages of plant-derived production systems for the synthesis of recombinant pharmaceutical proteins are widely discussed in a number of reviews [2,3,4,5]. In the context of biopharmaceutical manufacturing, the benefits of plant-derived systems include the ability to synthesize complex proteins with authentic post-translational modifications (e.g., glycosylation and disulfide bond formation), combined with low-cost upstream production, inherent process safety based on the inability of human pathogens to replicate in plants, and the potential for large-scale production [6,7,8,9]. The last two aspects are particularly important when comparing plants to mammalian cells, given the absence of contamination with human pathogens and the manufacturing capacity that can be rapidly adapted to meet market demands. A number of already commercialized products biomanufactured in plants were reviewed by Sack et al. [10].

Interferons are small, glycosylated polypeptides involved in the regulation and functions of the vertebrate immune system. Nowadays, the spread of numerous viral infections generates a high demand for cost-effective and scalable production of these biopharmaceuticals. Among the other forms of interferon, human IFN α-2b is widely used in medicine to treat specific malignancies and viral diseases, such as hepatitis [11]. SARS-CoV-2 caused a pandemic resulting in millions of deaths due to the immune system, inflammatory, and thrombotic response to the virus. Some multi-drug protocols for COVID-19 treatment include human IFN α-2b [12].

HuIFN α-2b has already been expressed in plants such as tobacco [13,14], potato [15], duckweed [16], carrot [17], endive [18], lettuce [18], aloe [19], rice [20], Jerusalem artichoke [21], canola [22], and radish [23]. Interferon production in tomatoes offers several advantages over these existing plant-based platforms. Crucially, tomato plants bear edible fruits that are free of toxic compounds. Moreover, lycopene—a powerful antioxidant carotenoid found in tomatoes—can enhance the overall response rate to antiviral therapy for hepatitis, delay disease progression, attenuate liver injury, and prevent the development of hepatitis C virus-induced hepatocellular carcinoma [24]. Although human interferons β and γ have already been expressed in two tomato species [25,26], there are no publications on the expression and biological activity of HuIFNα-2b in this genus.

The primary objective of this study was the demonstration of stable production and long-term preservation of biologically active recombinant human IFNα-2b in transgenic tomato fruits. Here we describe the genetic transformation of *Solanum lycopersicum* L. cv. “Shedevr 1”—a cultivar carrying the *alcobaca* (*alc*) gene for extended fruit shelf life—to express the human interferon α-2b gene sequence. High-level biological activity of the target protein in tomato tissues, its long-term stability during fruit storage, and transmission of transgenic traits to subsequent generations were confirmed.

## 2. Results

### 2.1. Genetic Transformation of Tomato and Molecular Analyses of Putative Transgenic Plants

**Figure 1:**
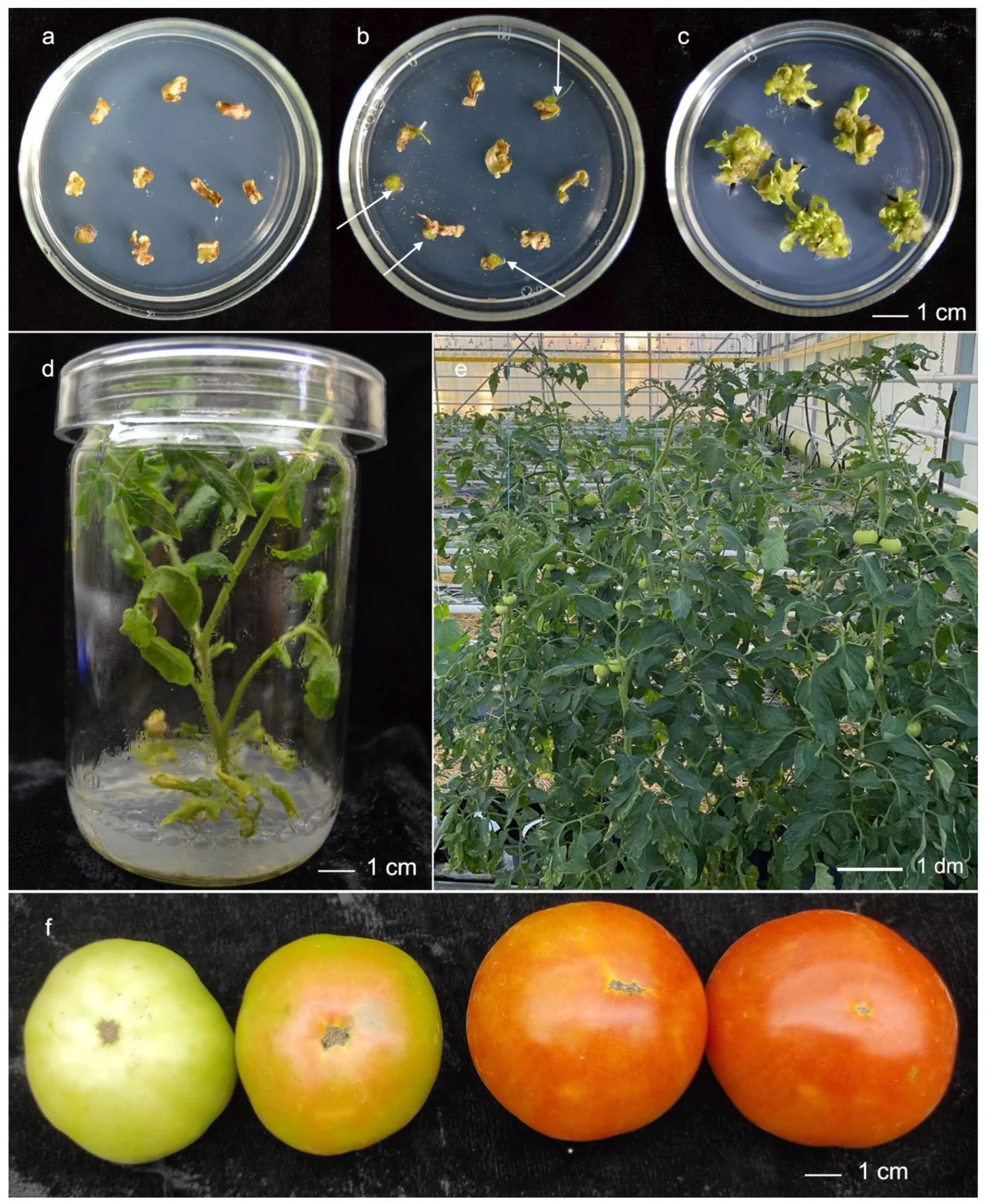
Stages of the production of transgenic tomatoes expressing HuIFN α-2b. Selection of transgenic tomato tissues on kanamycin-containing media (**a**–**c**): negative control showing tomato explants without *Agrobacterium* inoculation on a regeneration medium supplemented with 100 mg/L kanamycin (**a**); kanamycin-resistant tomato calli on regeneration medium with 100 mg/L of kanamycin after *Agrobacterium*-mediated transformation with pCB124 (**b**); positive control showing explants on the regeneration medium without kanamycin and *Agrobacterium* inoculation (**c**). A transgenic tomato plant grown in vitro (**d**). Greenhouse-grown transgenic tomato plants (**e**). Naturally ripened fruits of T_1_ transgenic tomato plants cv. ‘Shedevr 1’ (**f**). Arrows indicate emerging putative transgenic calli.

After cocultivation with *Agrobacterium*, cotyledon tissues cultured on the selective medium supplemented with kanamycin formed numerous green calli within 30–40 days, in contrast to the negative control (Figure 1a–c). Out of 100 inoculated cotyledon explants of cv. ‘Shedevr 1’, twenty-five kanamycin-resistant calli were obtained after 4 months of selection on MS medium with 100 mg/L of kanamycin. Shoot regeneration was observed in seven transgenic calli, resulting in a regeneration frequency of 7%. Only one shoot per callus was selected for further cultivation to ensure that each transgenic line originated from an independent transformation event. Excised shoots were rooted on the medium with 80 mg/L of kanamycin (Figure 1d). The transformation efficiency of cv. Lyana was lower, yielding twelve kanamycin-resistant calli, from which three putative transgenic shoots regenerated.

Transformed plants that survived the selection process were then screened for the *HuIFNα-2b* and *nptII* insertion in the tomato genome by PCR. Genomic PCR analysis confirmed the insertion of both genes into plant cells, as specific bands were detected in each transformant, whereas no bands were observed in an untransformed control. The *virC* gene fragment was absent in analyzed T_0_ plants. Furthermore, transcription of *HuIFNα-2b* gene in plants was confirmed by RT-PCR. After confirmation of the transgenic nature using molecular methods, T_0_ plants were transferred to the greenhouse for fruiting (Figure 1e,f).

### 2.2. Inheritance of Heterologous HuIFNα-2b and nptII Genes in T_1_ and T_2_ Generations of Transgenic Tomatoes

**Figure 2:**
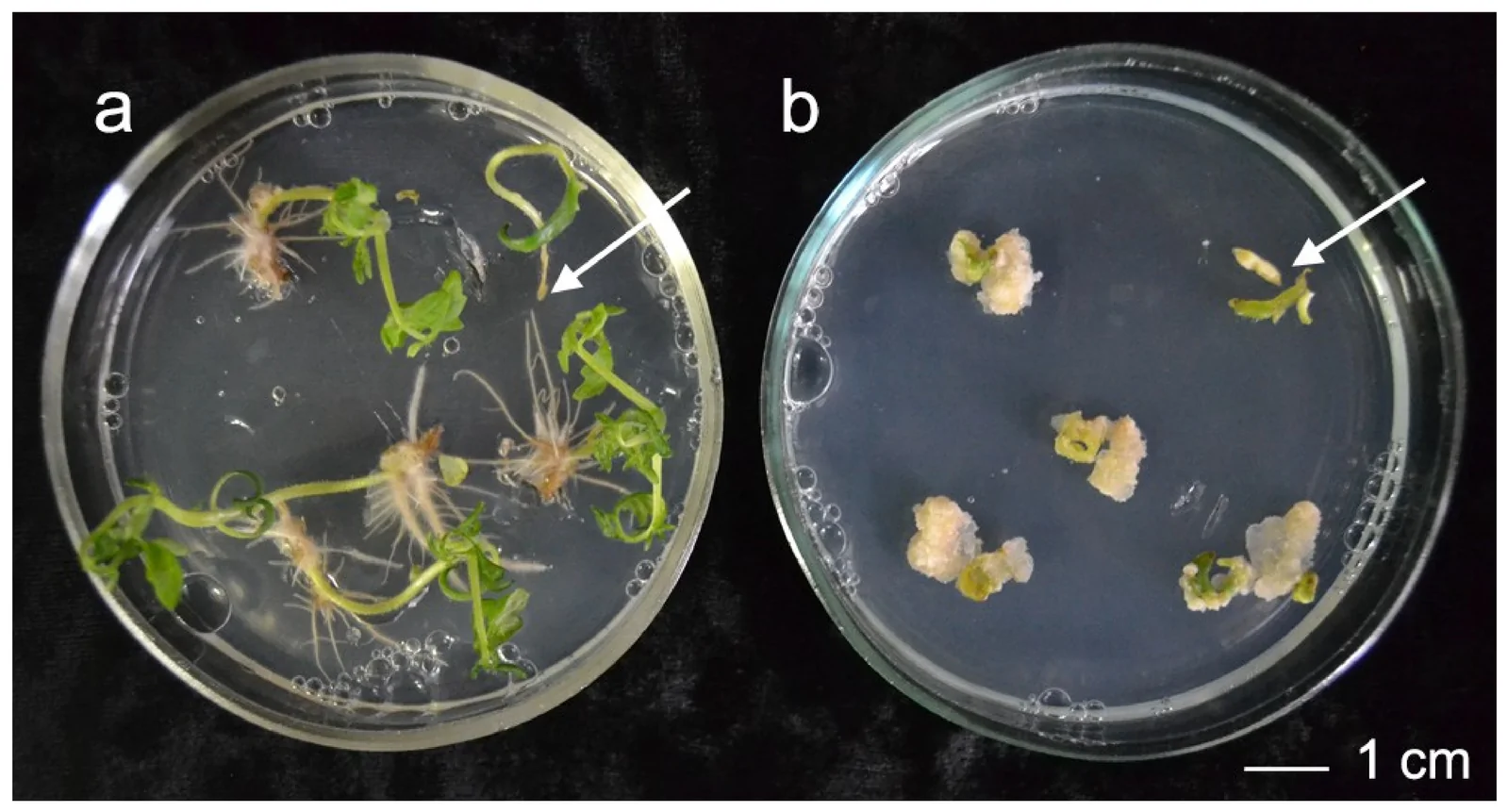
Segregation of T_1_ generation transgenic tomato plants on selective media. Rooting assay (**a**) and callus formation assay (**b**) on media supplemented with 100 mg/L kanamycin. The five shoots on the left Petri dish correspond to the respective cotyledon and hypocotyl explants on the right. Susceptible plants failing to form either roots or calli are indicated by arrows.

**Figure 3:**
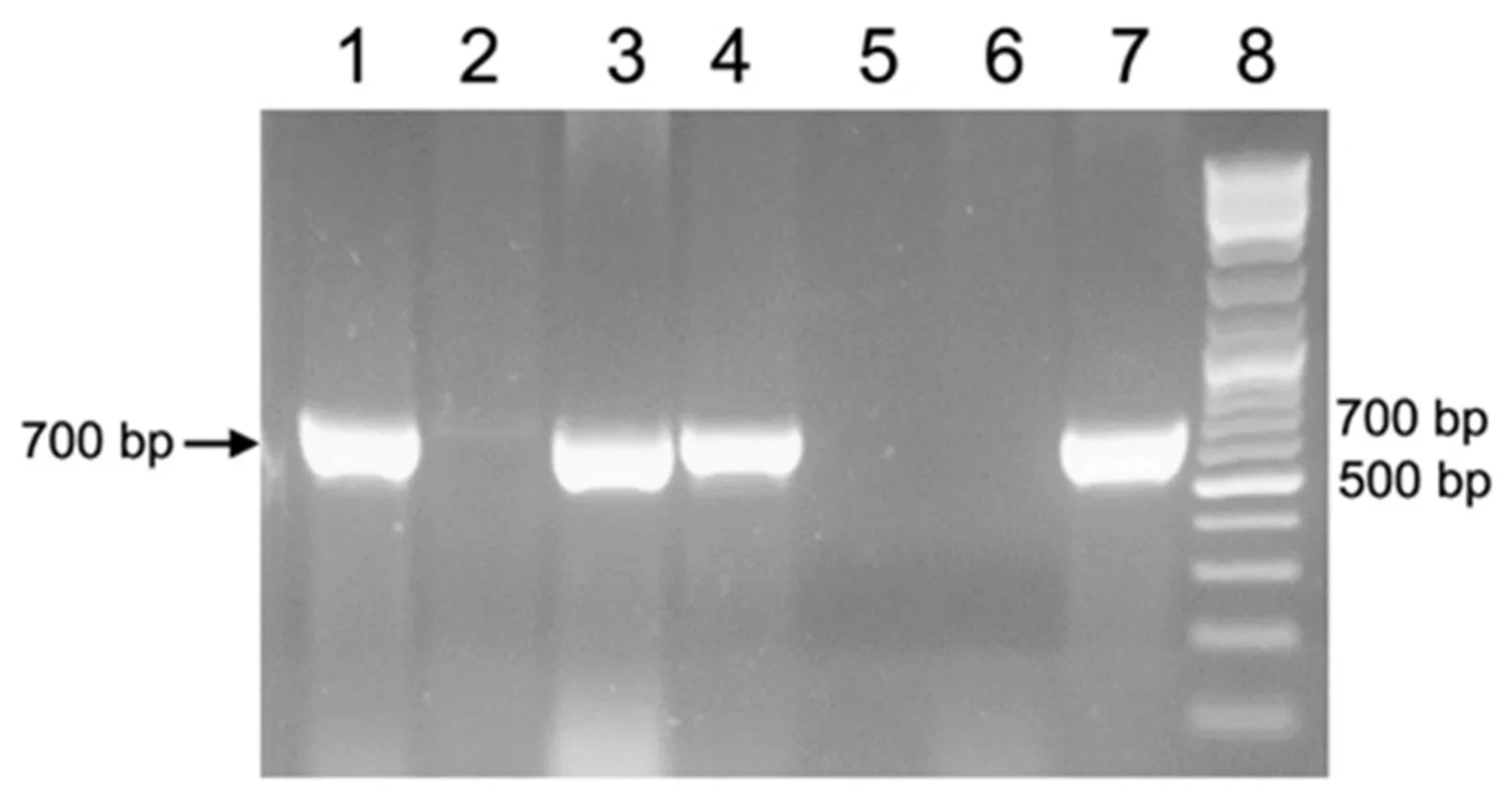
PCR analysis for the *nptII* gene fragment of the transformed tomato progeny germinated without kanamycin selection. Lanes 1, 3, and 4—DNA of transgenic T_1_ plants cv. ‘Shedevr 1’; Lane 2—segregated Km-sensitive offspring of T_0_ line No. 13; Lane 5—negative control (no template DNA); Lane 6—negative control (untransformed plant DNA); Lane 7—positive control (pCB124 plasmid DNA); Lane 8—DNA molecular weight marker (1 kb Plus DNA Ladder, Fermentas). The expected size of the amplicon is 700 bp.

**Figure 4:**
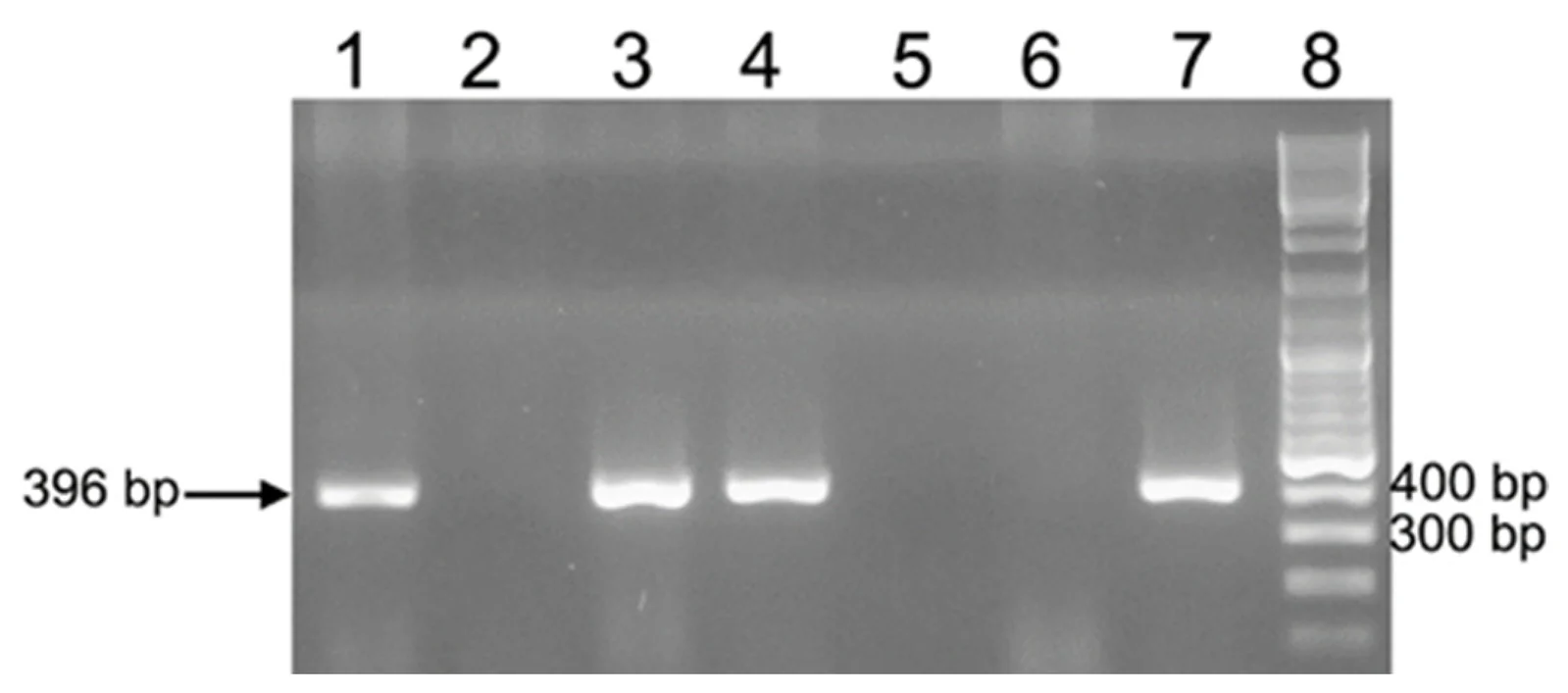
PCR analysis for the *HuIFNα-2b* gene fragment of the transformed tomato progeny germinated without kanamycin selection. Lanes 1, 3, and 4—DNA of transgenic T_1_ plants cv. ‘Shedevr 1’; Lane 2—segregated kanamycin-susceptible offspring of T_0_ line No. 13; Lane 5—negative control (no template DNA); Lane 6—negative control (untransformed plant DNA); Lane 7—positive control (pCB124 plasmid DNA); Lane 8—DNA molecular weight marker (1 kb Plus DNA Ladder, Fermentas). The expected size of the amplicon is 396 bp.

Seeds from the most vigorously growing transgenic T_0_ plants (three lines of cv. ‘Shedevr 1’ and one line of cv. ‘Lyana’) were collected upon harvest. Transgene inheritance and segregation patterns were determined in T_1_ transgenic plants of cv. ‘Shedevr 1’ by kanamycin resistance assay. Green plantlets forming roots and fast-growing calli were scored as kanamycin resistant (Figure 2). PCR analysis confirmed the presence of *nptII* gene in all the kanamycin-resistant plants. PCR analysis with primers to *HuIFN α-2b* gene revealed co-segregation of both genes in analyzed plants (Figure 3 and Figure 4).

According to the Chi-square (χ^2^) analysis of *nptII* gene segregation, we propose that the T-DNA integration occurred at a single genomic locus in the analyzed transgenic T0 tomato plants, showing a good fit to the expected 3:1 Mendelian ratio (*p* ≤ 0.05). 

The T_1_ − T_2_ plant generations were successfully grown in a greenhouse from T_0_ progenitors and analyzed by PCR for the presence of both *HuIFNα-2b* and *nptII*. PCR analysis of kanamycin-resistant T_1_ generation confirmed the presence of both *HuIFNα-2b* and the *nptII* genes. The transgenic progeny of lines No. 2, 4, and 13 were selected as representative examples for further analyses of T_2_ generation. The *HuIFNα-2b* and the *nptII* genes were stably transmitted across two subsequent generations of transgenic tomato plants.

PCR analysis of DNA from 268 T_2_ tomato seedlings (grown without kanamycin selection) revealed that 80.6% of seedlings of the analyzed line were kanamycin resistant and positive for *HuIFNα-2b* gene, indicating that the analyzed line was heterozygous. Additionally, homozygous lines showing no transgene segregation were identified.

### 2.3. Expression of HuIFNα-2b Gene in Transgenic Tomato Plants

**Figure 5:**
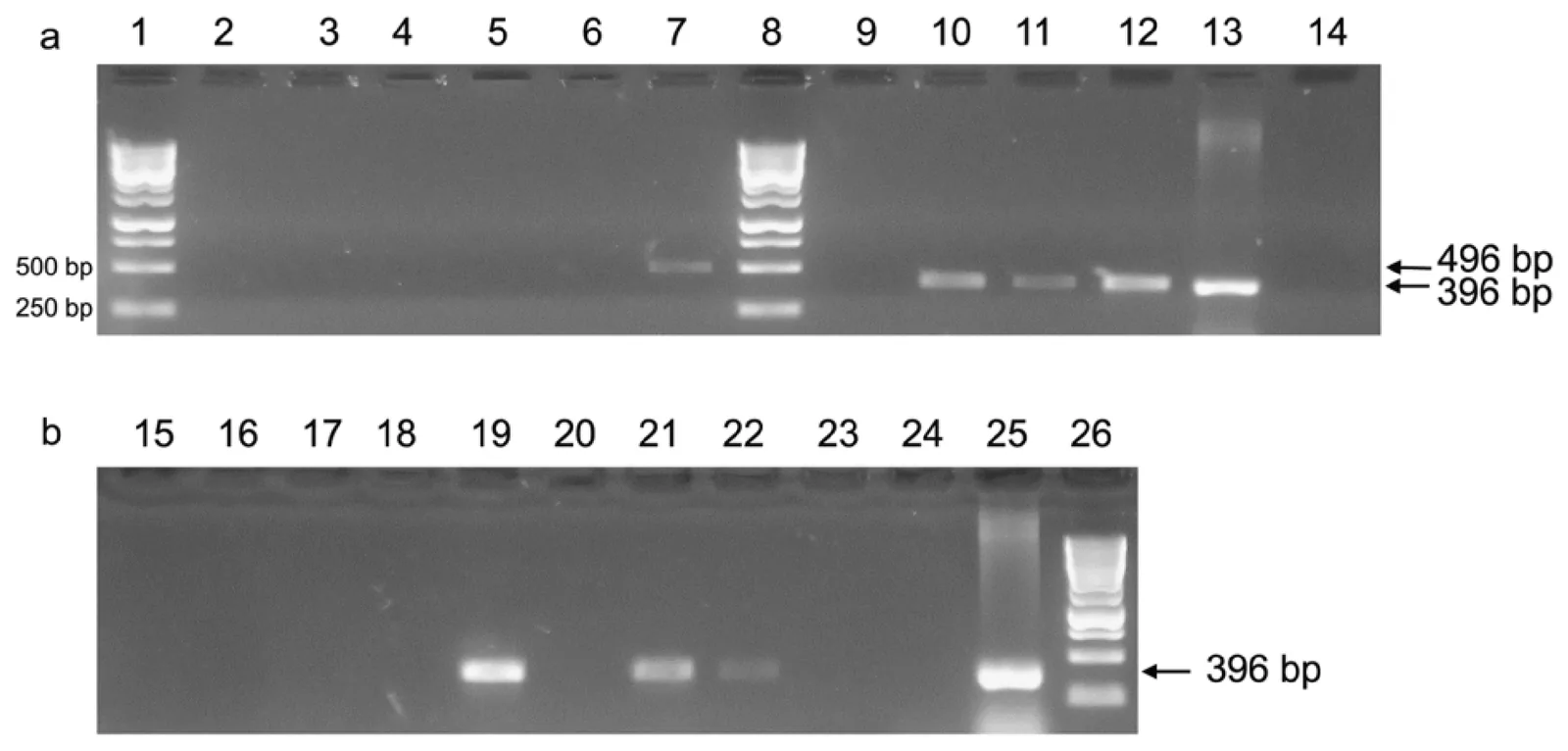
RT-PCR analysis of HuIFNα-2b gene expression in ‘Shedevr 1’ T_2_ transgenic tomato lines. Amplification products from plant leaves (**a**) and fruits (**b**). Lanes 1, 8, and 26—DNA marker ladder (Thermo Scientific GeneRuler1 kb, 250–10,000 bp); 2, 9, and 23—RNA (2) and cDNA (9, 23) from non-transgenic tomato (negative controls); Lanes 3–6—RNA from transgenic T_2_ lines (progeny of T_0_ ancestor No. 13); Lanes 10–13—corresponding cDNA from the same transgenic T_2_ lines; Lanes 4, 14, and 24—no-template control (reagent mix without cDNA); Lanes 7—*GAPDH* gene cDNA (496 bp, internal housekeeping control); Lanes 15, 19 and 17, 21—RNA (15, 17) and cDNA (19, 21) from two distinct T_2_ lines (progeny of T_0_ No. 13) after 2 weeks of fruit storage; Lanes 16, 20 and 18, 22—RNA (16, 18) and cDNA (20, 22) from the respective lines after 2 months of fruit storage; Lane 25—positive control (amplification of genomic DNA isolated from transgenic tomato).

Expression of *HuIFNα-2b* mRNA in the T_2_ generation was assessed by RT-PCR. Total RNA was extracted from the leaves and fruits of T_2_ progenies derived from transgenic tomato line No. 13 (Figure 5). Primers specific to mRNA allowed the synthesis of a total cDNA pool in a reverse transcription reaction. Subsequent PCR amplification of cDNA with *HuIFNα-2b-*specific primers confirmed the presence of the corresponding mRNA in specimens isolated from just-harvested and 12-day-stored tomato fruits. In contrast, cDNA was nearly undetectable in samples from tomatoes stored for 40 days (Figure 5b). These data demonstrate the efficient transcription of mRNAs from the heterologous *HuIFNα-2b* gene and the subsequent degradation of transcripts after 2-week storage of fruits.

RT-qPCR was conducted to quantify gene expression levels during the prolonged storage of fruits. This analysis was performed using the same RNA samples that were utilized for the semi-quantitative RT-PCR. Analysis of the data underlying Figure S1 confirms the validity of the standard curve construction. The regression equation and the coefficient of determination (R^2^ = 0.71), displayed directly on the graph, were obtained using standard methodology and reflect the dependence of the quantification cycle (Cq) on the dilution factor of the pooled sample, calculated separately for each of the two amplification series. For the analyzed dataset, the following parameters were obtained: PCR efficiency (E) = 1.71, ΔΔCt = 2.07), and the resulting fold-change in expression (E^ΔΔCt^) = 3.03. Accounting for the real amplification efficiency, the normalized expression level of the *HuIFNα-2b* gene (relative to the *β-tubulin* reference gene) in the experimental sample (tomato stored for 12 days) was approximately 3.03-fold lower than that in the calibrator (fresh tomato analyzed immediately after harvesting). Since tubulin was utilized as the reference (normalizing) gene, the increase in the Cq value for interferon is correctly interpreted as a specific downregulation of the *HuIFNα-2b* gene during storage, rather than a shift in reference gene expression, provided that the expression of the internal control remains stable. 

### 2.4. In Vitro Biological Activity of Plant-Produced Interferon

Although transcription of the inserted gene was successful, it was necessary to determine the biological activity of HuIFNα-2b expressed in the transgenic plants. HuIFNα-2b activity was not determined in T_0_ plants. Instead, analysis of biological interferon activity was conducted in the next generation of transgenic tomato cv. ‘Shedevr 1’ expressing the HuIFNα-2b gene. Since T_1_ plants could be homozygous or hemizygous, the strategy involved rapid screening of various T_0_ descendants. Only resistant plants derived from T_1_ seeds selected for the *nptII* on a kanamycin-supplemented medium were evaluated in the IFN activity assay to exclude non-transgenic segregants. We used pooling of nine fruits from three plants for each sample to minimize biological variation and reduce reagent consumption during large-scale screening.

The capacity of crude fruit and leaf extracts to reduce the viral cytopathic effect (CPE) on African green monkey epithelial cells was evaluated. Extracts were prepared from transgenic progeny of tomato lines Nos. 2, 4, and 13 alongside the corresponding wild-type cultivar ‘Shedevr1’. The in vitro antiviral activity of the plant-expressed recombinant protein was confirmed via inhibition of VSV CPE in the MA-104 cell line, whereas wild-type extracts exhibited no protective effects. As summarized in Table 1, the protective IFN activity in the HuIFNα-2b transgenic plants was verified. The biological interferon activity of extracts from lines No 4 and No 13 exceeded that from line No 2. The biological activities of interferon from fruits and leaves of transgenic ‘Lyana’ line were 0.4 × 10^3^ IU/g fresh weight (FW) and 1.1 × 10^3^ IU/g FW, respectively. Because our aim was to identify transgenic lines with the highest IFN activity, we selected the progeny of lines No 4 and No 13 for further investigation.

**Figure 6:**
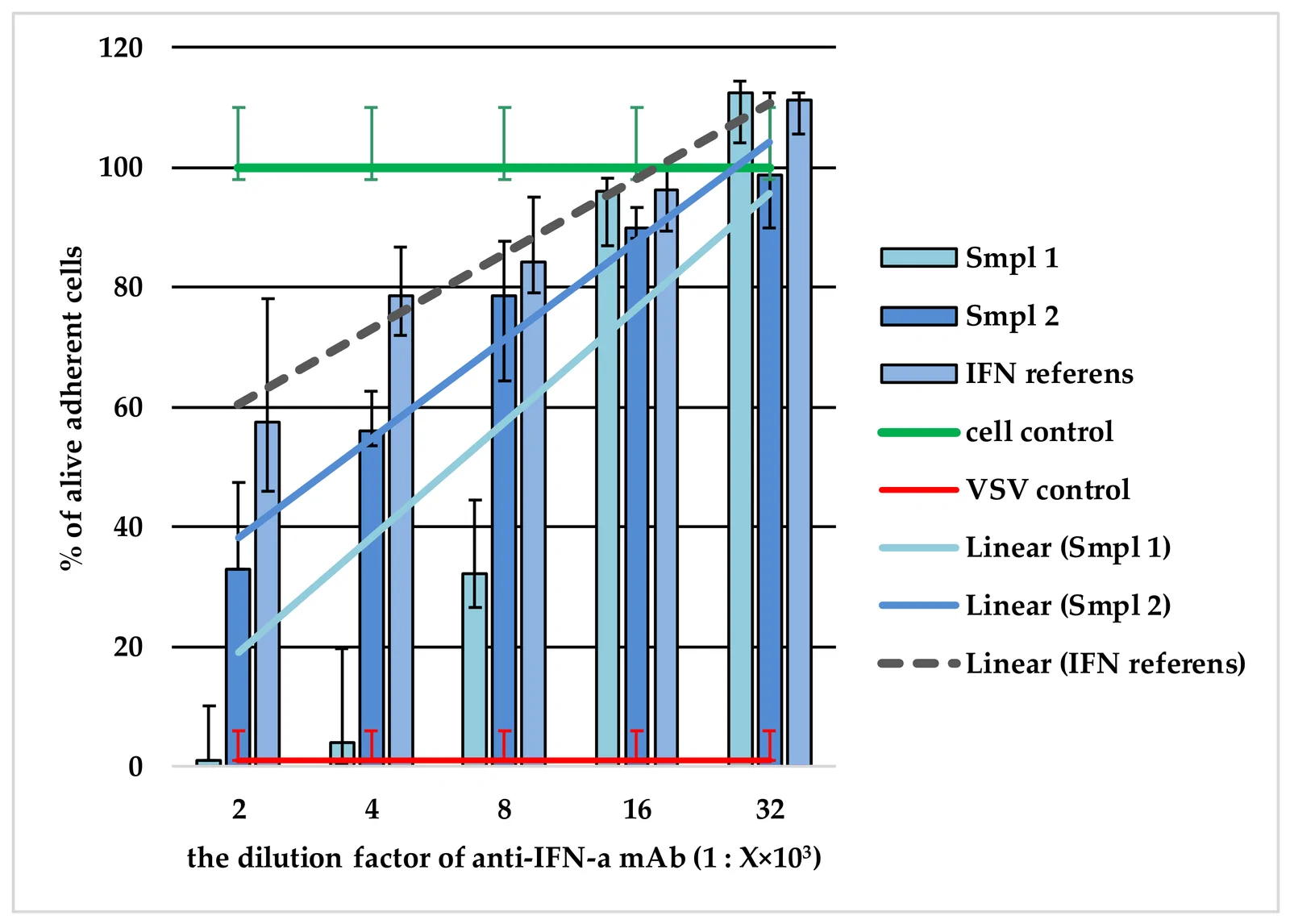
Neutralization curves (generated based on the original values of % of alive adherent cells) of leaf and fruit extract samples and of the HuIFN α-2b reference, obtained using the standard IFN method. The WHO International Standard for INTERFERON ALPHA 2b (Human rDNA derived, NIBSC code: 95/566) was used as the reference standard. The activity of the HuIFN α-2b reference sample before adding it to the anti-IFN-α mAb was 100 IU/mL, and the activity of the test samples (Smpl 1—leaves of the T_0_N13T_1_31 line, and Smpl 2—fruits of the T_0_N4T_1_3) in the analysis was similar, calculated based on the results of a preliminary determination of interferon activity within them. All samples were incubated with two-fold serial dilutions of anti-IFN-α mAb at a 1:1 (*v*/*v*) ratio at 37 °C for 1 h and added to the formed MA-104 monolayer for exposure to cells for 24 h. Subsequently, the cells were infected with VSV, and after 24 h, the presence/absence of the protective effect of interferon was determined. The viability lines of control cells (100% viability) and VSV-control (0% viability) are shown as controls. The lack of a protective effect in the reference HuIFN α-2b and leaf and fruit extract samples after exposure to minimal dilutions of anti-IFN-α mAb and its increase with increasing dilution of anti-IFN-α mAb indicate that the anti-IFN-α mAb neutralized interferon in the HuIFN α-2b reference and in the leaf and fruit extract samples.

To verify that the detected biological activity was explicitly driven by the heterologous protein, a neutralization assay of the extracts’ antiviral activity was performed using a monoclonal antibody (mAb) against HuIFNα-2b. This method is based on the principle that specific anti-interferon antibodies inactivate the target samples in a manner identical to the international standard for IFNα-2b. For this purpose, leaf and fruit plant extracts were randomly selected. Representative evaluation was conducted on leaf extracts derived from line T_0_ No. 13 (T_1_-31, Sample 1) and fruit extracts from line T_0_ No. 13 (T_1_-3, Sample 2). The findings are illustrated in Figure 6.

**Figure 7:**
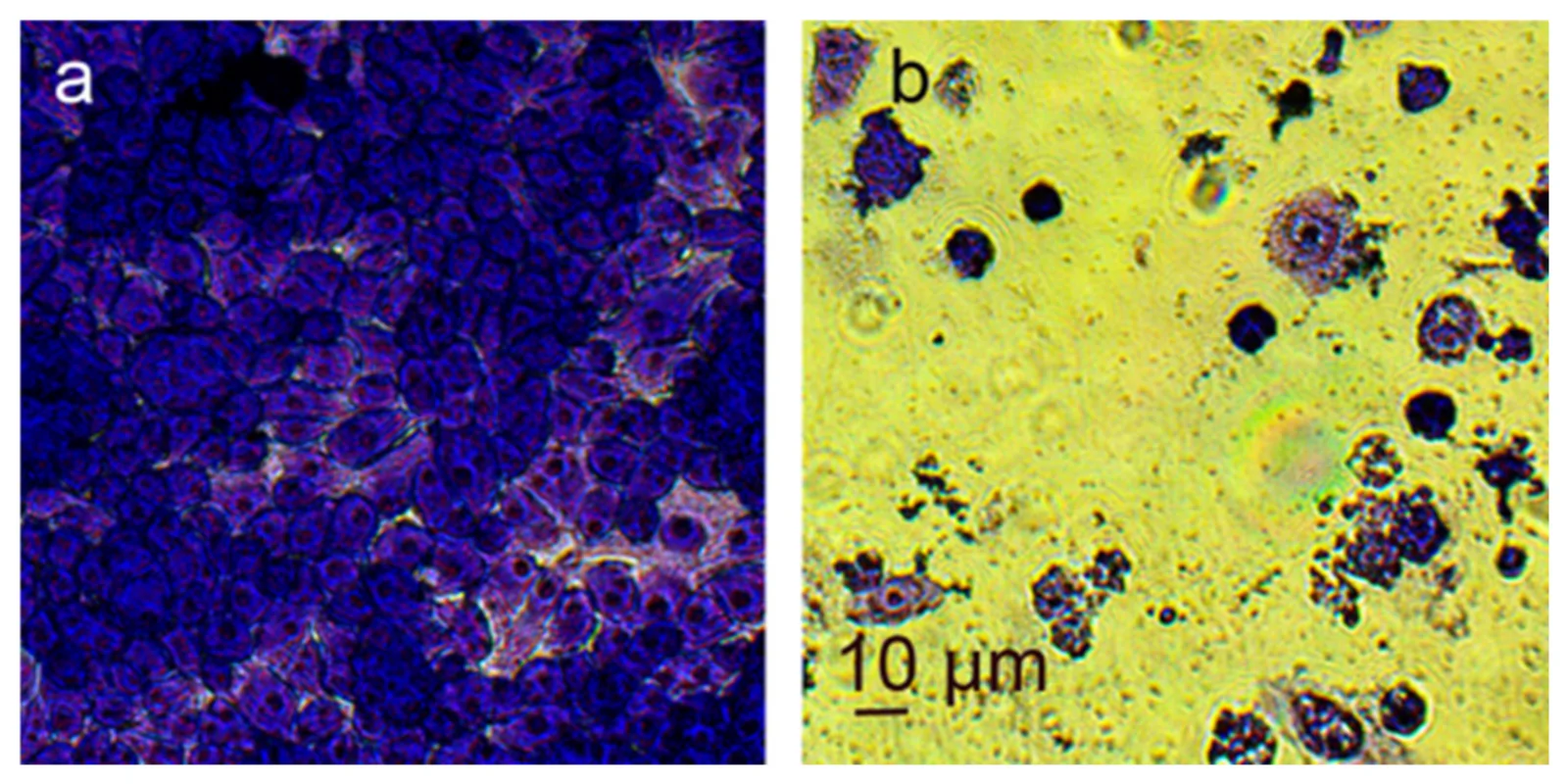
Morphology of intact epithelial kidney cell culture MA-104 from African green monkey (*Cercopithecus aethiops*) (**a**) and the cytodestructive effect of VSV on these cells (**b**). Magnification ×200.

**Figure 8:**
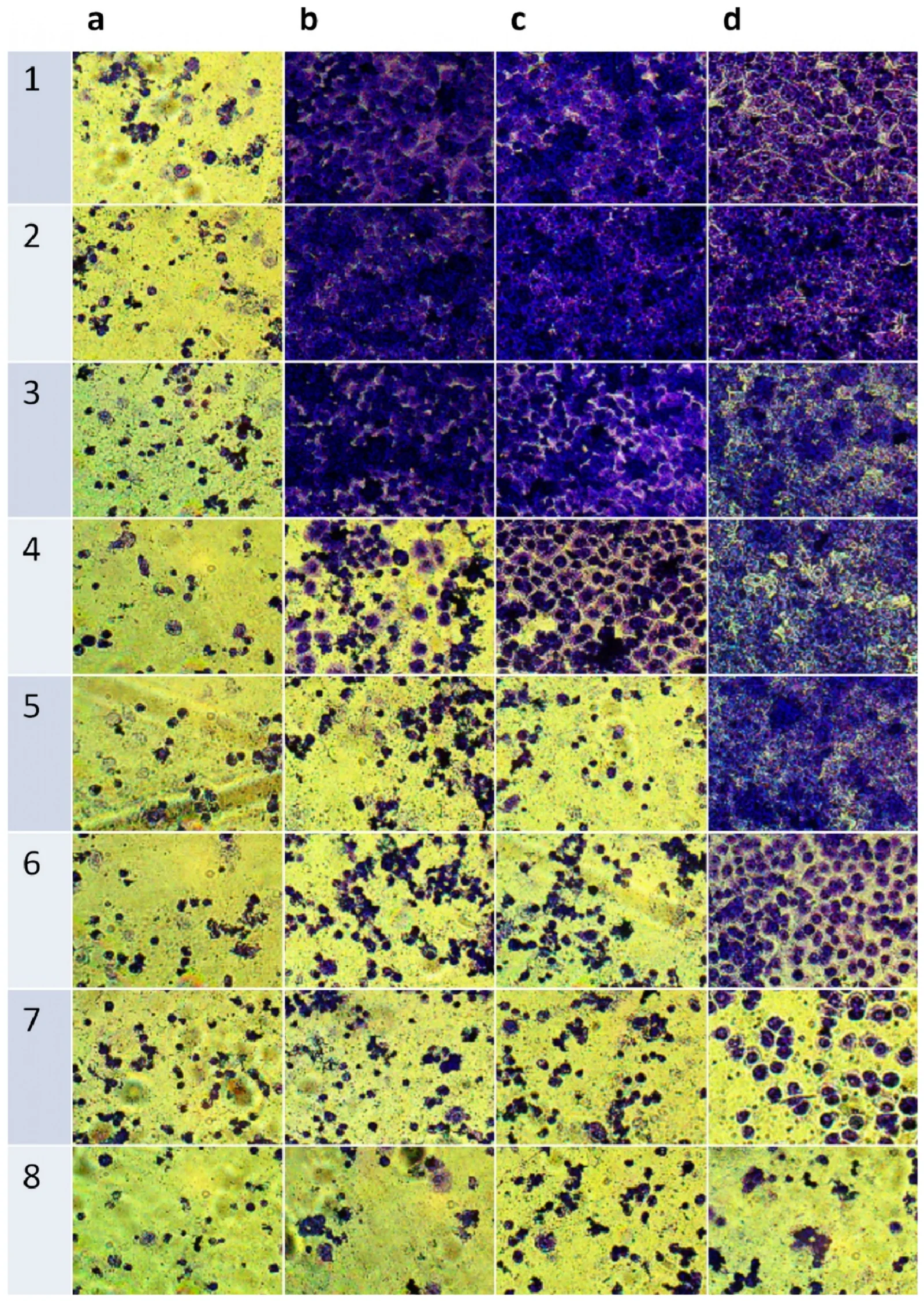
The protective effect of plant-based IFN-α2b. Monolayers of MA-104 cells were pretreated (for 24 h) with serial twofold dilutions (rows 1–8) of wild-type tomato extract (column a), two different samples of transgenic tomato extracts (columns b,c) and the WHO international standard for INTERFERON ALPHA 2b (Human rDNA derived, NIBSC code: 95/566) (column d) under conditions of VSV infection. Magnification ×200.

Intact cells form a confluent monolayer that can be stained with a crystal violet–ethanol solution, whereas the monolayer is disrupted if the cells are damaged by the cytopathic effect (CPE) of the virus. Bright-field microscopy of the cell monolayer confirmed the protective effect of extracts obtained from plants expressing the interferon gene compared to extracts from wild-type plants. An intact cell monolayer of the epithelial cell culture MA-104 Clone 1 (ATCC^®^ CRL-2378.1™) and the cytopathic effect of VSV (ATCC^®^ № VR-158) on these cells are demonstrated in Figure 7. Furthermore, the influence of the reference interferon (rDNA-derived WHO International Standard for HuIFN α-2b) and plant extracts (mock and from transgenic plants expressing the *HuIFNα-2b* gene) on the interaction of VSV with the MA-104 cell culture is shown in Figure 8. Recombinant interferon expressed in transgenic tomatoes carrying the *HuIFNα-2b* gene successfully inhibits VSV-induced cytodestruction in the MA-104 cell culture.

At harvest (Month 0), the initial biological interferon activity in the tested transgenic tomato fruit lines was 14,400 IU/g FW (range 13,900–14,900 IU/g FW). After long-term storage for three months, high biological interferon activity was retained in transgenic tomato fruits (4760 IU/g FW, range 2422–6720 IU/g FW). Subsequently, its activity decreased but was detected up to 5 months of fruit storage (350 IU/g FW).

Our data confirm the correct transcription and translation of the *HuIFNα-2b* gene in tomato plants and in vitro activity of interferon. The *HuIFNα-2b* gene retains its efficacy across generations under greenhouse conditions. No impact on plant growth, development or crop yield was observed (see Table S1 in the Supplementary Materials). These results demonstrate that biologically active human cytokine with potential pharmaceutical applications can be successfully expressed and stored in transgenic tomato plants.

## 3. Discussion

The highest level of interferon accumulation among the interferon-expressing plant systems was previously observed in transplastomic tobacco [13]. The level of IFN biological activity in obtained transgenic tomato fruits is within the range of or even exceeds other transgenic plant systems that have been used for the production of IFN, such as rice [20], duckweed [27], carrot [17], aloe [19], endive [18], lettuce [18], potato [15], Jerusalem artichoke [21] and canola [22]. Among stably transformed interferon expression systems [28], the obtained transgenic tomatoes demonstrated one of the highest biological interferon activities in tissues.

According to Gunasekaran, the major disadvantage of using tomato as a host plant for edible protein drug production is rapid fruit deterioration after ripening [5]. We used plants of *Solanum lycopersicum* L. cv. ‘Shedevr 1’, harboring mutant gene *alc*, which greatly prolongs shelf life, due to relatively low levels of endogenous ethylene and reduced activities of polygalacturonase and polymethylgalacturonase [29]. A few authors tested the storage time of tomato fruits with the *alc* gene for 30–40 days [29,30]. In our experiments, fruits of ‘Shedevr 1’ were stored for five months, and biological interferon activity was retained during this period in transgenic plants. RT-PCR analysis revealed that *HuIFNα-2b* gene expression ceased and levels of mRNA decreased after two weeks of storage. But interferon activity was detected even after five months of intact fruit storage. We propose that the protective effect of crude extracts from transgenic tomato lines against the cytopathic effect of VSV was maintained for such a long period due to the unique metabolic profile of ‘Shedevr 1’ harboring the *alc* gene. The fruits of the cultivar are usually gathered at the mature green stage and have delayed fruit deterioration under a low room temperature. According to [31], proteolysis in wild-type tomato is increased in on-vine ripened fruits stored at 4 °C compared to those ripened on shelf of a growing cabinet. Both transgenic fruits and leaves of early ripening cv. Lyana had much lower IFN activities compared to ‘Shedevr 1’. This can be related to the active metabolism of this early ripening cultivar and rapid protein degradation. The differences in amino acid metabolism between the common tomato fruits and those from long shelf-life mutants were confirmed by Osorio et al. [32].

The CaMV 35S promoter used in this study is not tissue-specific and remains the most widely utilized promoter for plant expression. Heterologous genes under the control of this promoter are expressed constitutively across all plant tissues [33], meaning that *HuIFNα-2b* driven by the CaMV 35S is expressed in all tomato organs. Gene expression was confirmed in both fruit and leaf tissues. The protective effect against viral cytopathic effect differed in crude extracts of tomato fruits and leaves. The levels of interferon activity in fruit and leaf tissues were expressed in IU of interferon activity per gram of FW and compared within the same plant clones harboring the *HuIFNα-2b* gene driven by the CaMV 35S. It was found that the biological interferon activity of leaf extracts exceeded that obtained from fruit tissues.

The transport of interferon facilitated by calreticulin apoplast targeting signal represents another putative mechanism for long-term retention of HuIFNα-2b antiviral activity. The recombinant human interferon α-2b gene (*HuIFNα-2b*) was fused with *Nicotiana plumbaginifolia* calreticulin apoplast targeting signal under the control of the CaMV 35S promoter. Proteins directed by this signal are targeted to specific subcellular compartments, including the Golgi apparatus, the endoplasmic reticulum, the plasma membrane, and cell walls [34,35]. Preferential localization of the target protein in the cell walls, outside the cytoplasm, indicates its involvement in the secretory pathway, which protects the protein from intracellular plant proteases. While our findings offer a plausible explanation for the long-term persistence of IFNα-2b in transgenic tomato fruits, further research is required to determine its precise subcellular localization and to provide direct biochemical evidence of its resistance to slow proteolysis.

The neutralization assay is particularly important because it demonstrates that the antiviral activity detected in transgenic tomato extracts possesses the antigenic determinants characteristic of authentic human IFNα-2b. Since endogenous tomato proteins are not recognized by these monoclonal antibodies, the marked loss of antiviral activity following antibody neutralization provides strong functional evidence that the detected biological activity is directly attributable to recombinant human interferon.

The titration curves of IFN activity neutralization in the transgenic tomato extracts from line T_0_N4T_1_3 and the reference sample of HuIFN α-2b are principally matched. The titration curve of the sample from T_0_N4T_1_3 was practically identical to that of the reference IFN. In contrast, the titration curve of the sample from T_0_N13T_1_31 was much steeper, asymptotically approaching the vertical line, which indicates a possible higher affinity of IFN present in the sample from T_0_N13T_1_31 toward the antibodies used. Also, the difference in the slope of the obtained titration curve may be related to the different affinity of the IFN present in the samples to the antibodies used in the work [36]. The differences detected may also be due to different specific activities of IFN in the samples, which may be associated with the specific conditions of sample preparation from leaves and fruits. The observed variations in IFN biological activity could be partially attributed to tissue-specific factors. The co-extraction of distinct endogenous pigments, such as chlorophylls from leaves and lycopene from mature fruits, as well as other co-extracted plant components, might influence the stability or interfere with the specific activity of the recombinant protein during downstream analysis. The observed differences may also reflect the variations in protein expression patterns across different plant organs. Overall, the data demonstrated that anti-human IFN-α mAbs are valuable tools to detect native IFNα-2b before further characterization. Complete neutralization of the antiviral effect by specific anti-HuIFNα-2b antibodies confirms that the observed cell protection is exclusively mediated by the recombinant target protein, ruling out false-positive interference from endogenous tomato metabolites. Thus, the neutralization reaction of extract activity proved that the protective antiviral activity is directly driven by plant-based HuIFNα-2b. The protective IFN biological activity of extracts from tissues of T_1_ and T_2_ generations demonstrated that the transgenic tomatoes continued to express the *IFNα-2b* gene and accumulated biologically active HuIFNα-2b. We also observed stable expression and transgene transmission across three successive tomato generations (T_0_–T_2_).

One of the major bottlenecks in plant molecular farming is the rapid degradation of recombinant proteins by endogenous host proteases, which often compromises the yield and post-harvest stability of biopharmaceuticals. In our study, the prolonged retention of HuIFNα-2b biological activity during fruit storage could be closely linked to the specific genetic background of the host matrix. We utilized the ripening-inhibited *alcobaca* (*alc*) tomato variant, a mutant well-documented for its significantly delayed senescence and downregulated profile of global hydrolytic and cell-wall-degrading enzymes during post-harvest storage. Since the overall metabolic breakdown and autolytic processes are severely diminished in *alc* fruits, it is highly plausible that this low-degradation microenvironment also suppresses non-specific proteolytic cleavage, thereby creating a protective matrix for the heterologous cytokine. Although direct profiling of protease kinetics and protein degradation assays were not performed in this work and remain a key objective for future studies, the genetically driven reduction in fruit autolysis provides a solid biological foundation for the hypothesis of observed stability of the recombinant interferon.

Transgenic, long-shelf-life tomatoes expressing *HuIFNα-2b* could serve as a viable platform for large-scale production of interferon formulated within lyophilized plant cells, standardized and encapsulated for oral administration. The long storage period without loss of activity facilitates a prolonged processing window in compliance with Good Manufacturing Practice (GMP) guidelines [37].

### Limitations of the Study

The main limitation of this study was the reliance on functional bioassays without direct physical visualization of the protein via Western blot. Furthermore, the exact biochemical mechanisms of interferon stability during tomato storage remain to be fully elucidated in future purification studies.

## 4. Materials and Methods

### 4.1. Plant Material

Plants of *Solanum lycopersicum* L. cv. ‘Shedevr 1’ harboring the mutant gene *alc* from abnormally ripening tomato mutant and early-ripening cv. Lyana were used in transformation experiments. Seeds of both cultivars were kindly provided by Dr. L.A. Rudas (Cherkasy State Agricultural Research Station of the National Scientific Center “Institute of agriculture of the national academy of agricultural sciences of Ukraine”).

### 4.2. Bacterial Strain

**Figure 9:**
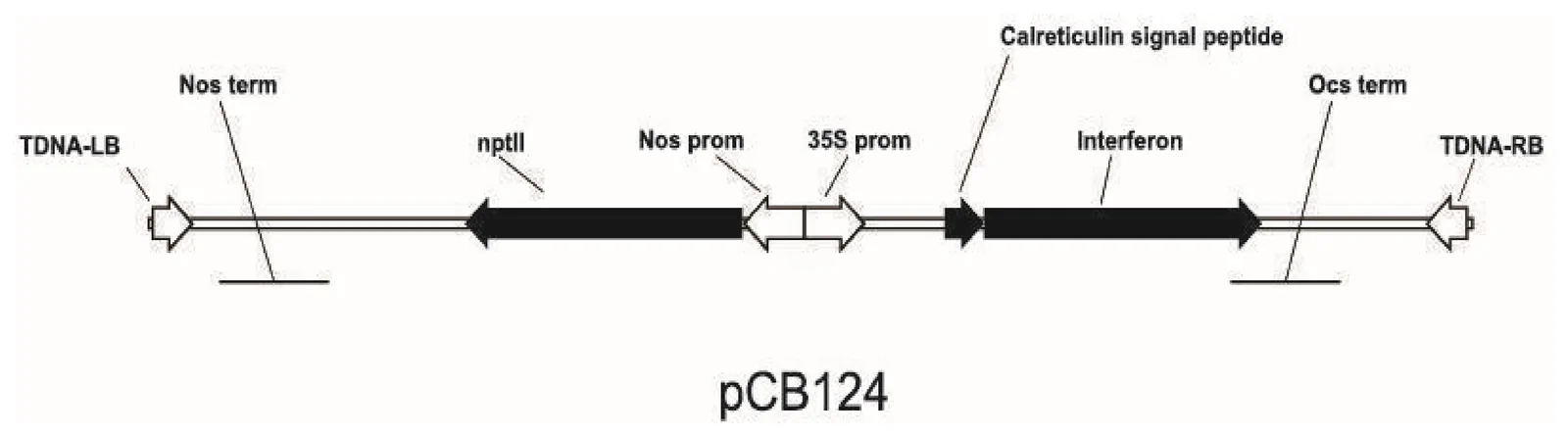
Scheme of T-DNA for pCB124. Schematic representation of the T-DNA region of the plasmid pCB124 used for tomato transformation. T-DNA-LB, left border of T-DNA; Interferon, human interferon α-2b gene (*HuIFNα-2b*); *nptII*, neomycinphosphotransferase gene; Calreticulin signal peptide, *Nicotiana plumbagenifolia* calreticulin apoplast targeting signal peptide; 35S prom, CaMV 35S promoter derived from the cauliflower mosaic virus; Nos prom, nopaline synthase (*nos*) promoter; Ocs term and Nos term, octopine synthase (*ocs*) and nopaline synthase (*nos*) terminators; T-DNA-RB, T-DNA right border.

Vector pCB124 in *Agrobacterium tumefaciens* nopaline strain GV3101 was used for tomato genetic transformation. Construction of the pCB124 plasmid was previously described [17]. Vector pCB124 contains the native human interferon α-2b gene (*HuIFNα-2b*) fused with *Nicotiana plumbaginifolia* calreticulin apoplast targeting signal under the control of the CaMV 35S promoter, as well as the selection gene for neomycin phosphotransferase II (*nptII*) driven by the nopaline synthase (nos) promoter (Figure 9).

The bacterial suspension was cultured overnight in liquid LB medium (10 g/L tryptone, 5 g/L yeast extract, and 10 g/L sodium chloride) [38] at 27 °C on a rotary shaker (150 rpm) supplemented with 50 mg/L rifampicin, 50 mg/L carbenicillin and 25 mg/L gentamicin.

### 4.3. Genetic Transformation 

Tomato seeds were surface-sterilized by dipping in 70% ethanol for 1 min, followed by 50% commercial bleach for 20 min under vacuum infiltration (up to 0.2 atm). The seeds were subsequently rinsed five times with sterile distilled water. Tomato seeds were germinated on half-strength MS (1/2MS) [39] with 20 g/L sucrose for 2–3 days in the dark at 27–28 °C, followed by 4–5 days under light conditions (24 °C).

Cotyledons were cut and injured with sterile blade 3–4 times and carefully transferred to callus induction medium (MS medium with 30 g/L sucrose, 1 mg/L thiamine HCl, 200 μM acetosyringone, 0.2 mg/L kinetin and 1 mg/L 2,4-D under dim light (24 °C) for 1 day.

The bacterial suspension culture was harvested by centrifugation (4000 rpm, 4 °C) and re-suspended in 10 mM MgSO_4_ solution. Cotyledons were transferred to empty Petri dishes, treated with the *Agrobacterium* suspension for 10 min, blotted with sterile filter paper and returned to callus induction medium for 2 days under the dim light. Then transformed cotyledons were transferred to shoot induction medium I (MS medium plus 1 mg/L thiamine HCl, 2 mg/L zeatin, 50 mg/L kanamycin as selective agent and 500 mg/L cefotaxime for *Agrobacterium* elimination) and kept in the growth room (2000 lk, 24 °C). Every 14–16 days, the cotyledons were subcultured onto fresh shoot induction tomato medium II (MS medium plus 1 mg/L thiamine HCl, 1 mg/L zeatin, 0.5 mg/L gibberellic acid (GA_3_), 500 mg/L cefotaxime and 100 mg/L kanamycin until green shoots were formed. Shoots 1–1.5 cm long were dissected and placed into root induction medium (1/2 MS supplemented with 20 g/L sucrose, 0.1 mg/L NAA, 250 mg/L cefotaxime and 50 mg/L kanamycin). Most of these shoots produced roots in 8–12 days. Then they were transferred to the 1/2 MS medium with 80 mg/L kanamycin and 250 mg/L cefotaxime. After 3 weeks, they were propagated and transferred to the greenhouse.

### 4.4. PCR, RT-PCR and qRT-PCR Assays

The presence of the transgene in the tomato plants was proved by PCR analysis. The total DNA was extracted from leaves using the CTAB method [40]. Total agrobacterial DNA was extracted according to the protocol described by Draper et al. [41]. The primer sequences for *HuINFa-2b*, *nptII,* and *virC* genes used for the PCR analysis are listed in Table 2. The primer pair for amplification of a 720 bp fragment of the *virC* gene was used to exclude bacterial contamination. 

Amplification of the *HuINFa-2b* gene fragment was carried out under the following conditions: 4 min at 94 °C, followed by 34 cycles of 30 s at 94 °C, 30 s at 60 °C, and 30 s at 72 °C, with a final extension for 5 min at 72 °C. The samples were fractionated in a 1.2% agarose gel in an LB buffer (composed of 10 mM lithium hydroxide and 35 mM boric acid) [45]. Synthesis of the *nptII* gene fragment was carried out under the following conditions: 4 min at 94 °C, followed by 9 cycles of 30 s at 94 °C, 45 s at 68 °C, and 30 s at 72 °C, followed by 25 cycles of 30 s at 94 °C, 30 s at 60 °C, and 30 s at 72 °C, with a final extension for 5 min at 72 °C. The amplification conditions for the *virC* fragment were: 3 min at 94 °C, followed by 40 cycles of 30 s at 94 °C, 47 s at 55 °C, and 30 s at 72 °C, with a final extension for 5 min at 72 °C. The samples were fractionated in a 1% agarose gel in the LB buffer.

Total RNA was extracted from fruits (either newly harvested or stored for 12 days and two months) and freshly collected leaves to estimate *HuIFNα-2b* expression. Total RNA was isolated using the RNeasy Mini Kit (QIAGEN, Hilden, Germany). The High-Capacity cDNA Reverse Transcription Kit (Thermo Fisher Scientific, Waltham, MA, USA) was used for cDNA generation according to the manufacturer’s manual. Each sample was analyzed with and without the addition of M-MuLV reverse transcriptase. Reverse transcriptase PCR analysis RT-PCR) amplification was conducted as described above. The reaction mixtures were analyzed by electrophoresis on a 1.2% agarose gel in an LB buffer. RNA isolation, cDNA synthesis, and RT-PCR analysis were performed at least in duplicate for each sample. 

Total RNA extraction was performed using the QIAGEN RNeasy Mini Kit (Cat. No. 74104) for RT quantitative PCR (RT-qPCR). Reverse transcription was carried out using the Thermo Fisher High-Capacity cDNA Reverse Transcription Kit (Cat. No. 4368814). RT-qPCR amplification was conducted using the Thermo Fisher Maxima SYBR Green/ROX qPCR Master Mix (2X) (Cat. No. K0221). The concentration and purity of the isolated nucleic acids were assessed using a NanoDrop spectrophotometer. Amplification and fluorescence detection were performed on a Bio-Rad CFX96 Touch Real-Time PCR Detection System. The relative expression levels were calculated sequentially according to the following equations:

ΔCq = Cq(target gene: *HuINFa-2b*) − Cq (reference gene: β-tubulin);

ΔΔCq = ΔCq(experimental sample) − ΔCq(calibrator);

In this study, sample No. 1 (fresh tomato prior to storage) served as the calibrator. The fold change in gene expression was determined as E^ΔΔCq^, where E represents the experimentally determined amplification efficiency (rather than the idealized value of 2, which assumes a 100% product doubling efficiency per cycle. The amplification efficiency (E) was calculated from the slope of the linear regression of Cq values plotted against the log_10_ of the serial dilution factor, using the following equation:

E = (10^−1/slope^ − 1) × 100%

The difference in amplification efficiencies between the target gene and the reference gene did not exceed 5%, thereby fulfilling the mandatory prerequisite for the correct application of the comparative ΔΔC_t_ method in relative quantification (Supplementary Materials, Figure S1) [46].

### 4.5. Segregation of Transgenes in T_1_ Plants

Fruits of the T_0_ plants transformed with pCB124 (and confirmed by PCR) were collected, and the isolated seeds were dried and stored in a cold chamber. Plantlets of the T_1_ generation were subjected to kanamycin resistance tests to eliminate segregated plants. Seeds of the T_1_ generation were sterilized and germinated as described previously. In 7–8-day-old seedlings, the roots were excised, and the shoot tips with one cotyledon were placed on a 1/2 MS medium supplemented with 100 mg/L kanamycin and 0.1 mg/L NAA for the rooting test. The excised cotyledons and hypocotyls were placed onto a callus induction medium (MS medium containing 30 g/L sucrose, 1 mg/L thiamine HCl, 0.2 mg/L kinetin and 1 mg/L 2,4-D with 100 mg/L kanamycin) to evaluate their capacity for resistant callus formation. Resistant T_1_ plants were further grown in 15 cm plastic pots containing (*v*/*v*/*v*) (soil:peat:sand) mixture. The presence of *nptII* and *HuIFNα-2b* genes in the resistant plants was confirmed by PCR. Subsequently, the T_2_ generation was analyzed in the same way.

### 4.6. Interferon Activity Assay

The extracts of tomato fruits (freshly harvested, as well as those stored for 3 and 5 months at 16–18 °C) and fresh leaves were prepared by homogenization in a double volume of extraction buffer containing 100 mM Tris-HCl (pH 8.0), 5 mM Na_2_EDTA, 100 mM NaCl, and 10 mM β-mercaptoethanol, supplemented with 2.5% polyvinylpyrrolidone. This was followed by a multi-step centrifugation process (10,000 rpm for 5–7 min, and 15,000 rpm for 25 min at 4 °C). For each independent transgenic line, a composite sample was prepared by pooling three representative fruits harvested from each of three individual plants. Plants derived from T_1_ seeds were grown on a kanamycin-supplemented medium to select for individuals containing the T-DNA insertion with *nptII* and linked *HuIFN* prior to IFN detection, thereby eliminating segregated null plants. The selected plantlets were vegetatively propagated in vitro, and six resulting plants per tested T_1_ line were subsequently transplanted into soil. Between two and four T_1_ lines were used to characterize each T_0_ line. Fruits at the stage of technical maturity and fully expanded leaves were used for extraction. The prolonged maintenance of biological IFN activity was evaluated in tomato fruits gathered at technical maturity and stored at room temperature (16–18 °C) for three and five months.

The total soluble protein (TSP) content in the plant crude extract was determined by the Bradford assay [47]. Bovine serum albumin (BSA, Sigma Chemical, St. Louis, MO, USA) was used as a standard in concentrations ranging from 0.05 to 0.5 mg/mL. Absorbance was read at a wavelength of 595 nm.

The interferon activity was measured by a microtitration method [48] based on the ability of the studied extracts to protect renal epithelial cells (line MA-104) from *Cercopithecus aethiops* (obtained from the cell collection of the D.K. Zabolotny Institute of Microbiology and Virology, of the NAS of Ukraine) against the cytopathic effect of the vesicular stomatitis virus (VSV), Indiana strain, also from the collection of the D.K. Zabolotny Institute of Microbiology and Virology, NAS of Ukraine. A 0.2 mL cell suspension (2 × 10^5^ cell/mL) was added to each well of 96-well plates (Falcon, USA) to form a cell monolayer under a stable CO_2_ level. The studied samples (0.2 mL) were added to the cell culture (using a two-fold serial dilution series in the well rows) after 24–48 h. Each sample dilution for IFN titration was analyzed in three technical replicates. After 18–20 h of incubation, the studied samples were removed, and 0.1 mL of the test-virus culture (100 TCD_50_) in a freshly prepared maintenance medium was added. The cell culture medium alone was used as a cell control, while the cell monolayer containing the indicated virus dose served as the virus control. The cells were stained with a 0.2% crystal violet solution in ethanol [49], followed by measurement of the viable cell quantity. The optical density (OD) of the stained cells was measured using a Multiskan spectrophotometer (UK) at a wavelength of 540 nm. The protective effect of interferon against the viral cytopathic effect was calculated as a ratio of the quantity of viable cells to uninfected control ones, considering the cytopathic effect in the virus control wells [50]. The activity units of the sample were calculated based on the highest dilution protecting 50% of the cells. The results obtained were validated only in the case of absence of cytodestructive changes in the cell control samples and upon complete cell degeneration in the virus control ones. The maximum sample dilution able to inhibit the cytopathic virus effect was considered as the interferon titer. Recombinant human interferon (rhIFNα-2b) from the WHO International Standard was used as a positive control. The human rhIFN-derived preparation (NIBSC code: 95/566) served as a reference standard (WHO/BS/2020.2389). The activity of the reference sample was 1000 IU/mL. According to established methodological frameworks [51,52], this approach provides high precision and reliability for biological quantification. The use of the WHO International Standard, assayed simultaneously with all experimental samples, effectively compensates for inter-assay variability associated with differences in cell susceptibility, virus infectivity, and experimental conditions. Therefore, the biological activity is expressed as relative potency in International Units (IUs) rather than as an absolute endpoint titer, thereby ensuring the high accuracy, reproducibility, and comparability of the results among different laboratories and experimental systems [51,52].

The interferon activity was expressed in international units (IUs) per gram of tomato leaf or fruit fresh weight (FW).

### 4.7. Bright-, Field Microscopy of MA-104 Cell Monolayers

The cell culture morphology and the viral cytopathic effect in wells containing mock controls or IFN dilutions were examined in the 96-well microplates with a bright-field microscope (Zeiss, Axiophot, Oberkochen West Germany). Images were captured using an M3CMOS 10,000 camera (Sigeta, Kyiv, Ukraine) and processed using ToupView software (ToupTek ToupView 3.3.10121). For microscopy, the microplates were inverted.

### 4.8. Interferon Neutralization Assay Using Anti-HuIFNα Monoclonal Antibodies

Samples of plant extracts were assayed for their susceptibility to inactivation by anti-*HuIFNα* -2b (mAbs), comparable to the WHO International Standard for HuIFNα-2b. A monoclonal mouse IgG_1_ antibody (clone MMHA-2, R&D Systems, Minneapolis, MN, USA), which neutralizes HuIFNα-2b and binds to it with high affinity (KA ≈ 10^9^ M^−1^), was employed.

The “standard-IFN method” was applied, where an equal amount of IFN was added to serial dilutions of anti-HuIFNα-2b mAbs to obtain a linear regression curve [53]. A series of five two-fold serial dilutions of the mouse anti-HuIFNα mAbs was prepared in a 24-well microplate. Two plant extract samples with previously determined activity were used: leaves of the T_0_N13T_1_31 line (140 × 10^3^ IU/mL) and fruits of the T_0_N4T_1_3 (4.96 ×10^3^ IU/mL) and WHO International Standard for HuIFNα-2b. The obtained dilutions of anti-HuIFNα-2b mAbs were mixed with the tested samples containing 100 IU/mL interferon at a 1:1 (*v*/*v*) ratio and carefully pipetted. The microplates containing mAb-sample mixtures were incubated in a CO_2_ incubator for 1 h at a temperature of (37 ± 2) °C, a carbon dioxide content of (5.0 ± 0.5)%, and a relative humidity of (70 ± 5)%.

The neutralization efficiency of the IFN antiviral activity in the tested samples was evaluated biologically in a passaged culture of interferon-sensitive MA-104 cells in the presence of the vesicular stomatitis virus (VSV) indicator strain, as described above. Specifically, 100 μL of each dilution (*n* = 4) was added to the wells, while cell culture medium alone was added to the control wells. After 24 h, the medium was removed, and 100 TCD_50_ VSV was added to the wells, except for the cell control wells, where maintenance medium was added instead. Development of the viral cytopathic effect (CPE) was observed 24 h after infection of the cell monolayer. Samples were considered to have passed the identification test if their antibody titration curves closely coincided with the titration curve of the IFN standard, provided that destruction of the cell monolayer by the virus was observed in the VSV-control wells, and no cytopathic effect occurred in the cell control wells.

### 4.9. Statistical Analysis

Segregation data were analyzed by the chi-square (χ^2^) test. Chi-square analysis for the genotypic and phenotypic ratio was carried out using the formula, χ^2^ = (O − E)2/E, where O is the observed value, and E is the expected value. For the single-gene model, each chi-square value was nonsignificant (*p* ≤ 0.05) if its value was greater than 3.84.

Statistical analysis using non-parametric methods was performed to evaluate the experimental data obtained from the assessment of plant-derived interferon antiviral activity. Continuous variables are presented as median and interquartile ranges (IQR) and as median (minimum–maximum) for datasets with a limited number of observations (*n* = 3). The program STATISTICA 10.0 (v. 13.3.721) was used.

## 5. Conclusions

The transgenic tomato plants obtained in this study demonstrated the potential of long-shelf-life tomato cv. ‘Shedevr 1’ as a plant-based system for the production and accumulation of biologically active HuIFNα-2b. The recombinant interferon accumulated in edible fruits and retained high biological activity for at least three months when the fruits were stored at moderate room temperature. The *HuIFNα-2b* transgene was stably inherited and expressed in subsequent generations of transgenic plants. These results indicate that the long-shelf-life tomato cultivar ‘Shedevr 1’ may provide a suitable platform for the stable production and storage of recombinant human interferon.

## Figures and Tables

**Table 1:** Average levels of HuIFNα-2b biological activity in fruit (pulp without seeds) and leaf extracts from T_1_ tomato plants derived from different primary transgenic lines of cv. ‘Shedevr 1’.

T_0_ Line		Fruits		Leaves	
		FW IU/g	TSP IU/mg	FW IU/g	TSP IU/mg
No. 2	Mean	2200	6100	67,900	3300
	Range	[800–3700]	[2700–9800]	[39,400–127,300]	[2000–4900]
No. 4	Mean	14,400	31,200	114,600	18,400
	Range	[13,900–14,900]	[21,000–41,300]	[82,700–146,400]	[4200–32,500]
No. 13	Mean	6600	11,900	389,300	22,900
	Range	[3100–10,100]	[10,300–13,400]	[139,600–854,000]	[5300–41,900]
Wild type	Mean	0	0	0	0

IU—international unit of interferon, FW—fresh weight, TSP—total soluble protein.

**Table 2:** Primers used for the PCR assays.

Gene	Primers	Expected Amplicon Size	Reference
*HuINFa-2b*	5′-TTC TGC TCT GAC AAC CTC-3′ 5′-TTG ATG CTC CTG GCA CAG-3′	396 bp	-
*nptII*	5′GAG GCT ATT CGG CTA TGA CTG–3′ 5′-ATC GGG AGC GGC GAT ACC GTA-3′	700 bp	[42,43]
*virC*	5′-ATC ATT TGT AGC GAC T-3′ 5′-AGC TCA AAC CTG CTT C-3′	720 bp	[44]

## Data Availability

The raw data supporting the conclusions of this manuscript will be made available by the authors, without undue reservation, to any qualified researcher upon request.

## Supplementary Materials

The following supporting information can be downloaded at https://www.mdpi.com/article/10.3390/plants15182849/s1, Table S1: Comparative morphological and phenotypic characteristics of non-transgenic and transgenic lines of the ‘Shedevr 1’ tomato variety. Figure S1: Standard curve for amplification efficiency evaluation.

## Abbreviations

The following abbreviations are used in this manuscript:

FW	Fresh weight
CPE	Cytopathic effect
GA_3_	Gibberellic acid
GMP	Good Manufacturing Practice
HuIFNα-2b	Human interferon α-2b (protein)
*HuIFNα-2b*	Gene of human interferon α-2b gene
IU	International unit
LB buffer	Lithium borate buffer
LB medium	Lysogeny broth medium
mAbs	Monoclonal antibodies
NAA	1-Naphthaleneacetic acid
TCD	Tissue cytotoxic dose
TSP	Total soluble protein
VSV	Vesicular stomatitis virus
