# Supplementary material for: Stability of Biologically Active Human Interferon α-2b in Transgenic “Long-Shelf Life” Tomatoes and Their Progeny
Source: Plants (Basel). 2026 Sep 17;15(18):2849. doi: 10.3390/plants15182849 (PMC13611584; doi:10.3390/plants15182849)
Supplement: Supplementary file 1 [file plants-15-02849-s001.zip › plants-4461883-supplementary.pdf]

Supplementary Material

Table S1. Comparative morphological and phenotypic characteristics of non-transgenic and transgenic lines of the 'Shedevr 1' tomato variety.

№	Morphological trait / Phenotypic expression	Analyzed plants				
		Non-transgenic plants	Line N2 T_1 N6	Line N4 T_1 N3	Line N4 T_1 N4	Line N 13 T_1 N 31
1	Hypocotyl coloration	Green-violet	Green-violet	Green-violet	Green-violet	Green-violet
2	Cotyledon shape	Round-lanceolate	Round-lanceolate	Round-lanceolate	Round-lanceolate	Round-lanceolate
3	Cotyledon length	Medium (2 –3 cm)	Medium (2 –3 cm)	Medium (2 – 3 cm)	Medium (2 –3 cm)	Medium (2 –3 cm)
4	Cotyledon width	Medium (0.5 – 1.0 cm)	Medium (0.5 – 1.0 cm)	Medium (0.5 – 1.0 cm)	Medium (0.5 – 1.0 cm)	(0.5 –1 cm) (0.5 – 1.0 cm)
5	Cotyledon coloration	Green	Green	Green	Green	Green
6	Hypocotyl length	Medium (2 –3 cm)	Medium (2 –3 cm)	Medium (2 – 3 cm)	Medium (2 –3 cm)	Medium (2 –3 cm)
7	Root system type	Fibrous	Fibrous	Fibrous	Fibrous	Fibrous
8	Rooting depth	Shallow	Shallow	Shallow	Shallow	Shallow
9	Plant growth habit	Determinate	Determinate	Determinate	Determinate	Determinate
10	Plant shape	Prostrate (creeping)	Prostrate (creeping)	Prostrate (creeping)	Prostrate (creeping)	Prostrate (creeping)
11	Plant branching	Medium (4 –8)	Medium (4 – 8)	Medium (4 – 8)	Medium (4 –8)	Medium (4 –8)
12	Shoot formation pattern	At the base of the plant	At the base of the plant	At the base of the plant	At the base of the plant	At the base of the plant
13	Foliage density	Medium	Medium	Medium	Medium	Medium
14	Main stem height	Short (30 – 50 cm)	Short (30 –50 cm)	Short (30 –50 cm)	Short (30 –50 cm)	Short (30 –50 cm)
15	Internode length	Medium (5 –10 cm)	Medium (5 – 10 cm)	Medium (5 – 10 cm)	Medium (5 –10 cm)	Medium (5 –10 cm)
16	Internode surface	Smooth	Smooth	Smooth	Smooth	Smooth
17	Stem pubescence	Medium	Medium	Medium	Medium	Medium
18;	Leaf type	Standard (regular)	Standard (regular)	Standard (regular)	Standard (regular)	Standard (regular)
19	Leaf size	Medium (15 –25 cm)	Medium (15 – 25 cm)	Medium (15 –25 cm)	Medium (15 –25 cm)	Medium (15 –25 cm)
20	Leaf coloration	Dark green	Dark green	Dark green	Dark green	Dark green
21	Leaf color pattern	Glossy	Glossy	Glossy	Glossy	Glossy
22	Leaf venation	Weakly expressed	Weakly expressed	Weakly expressed	Weakly expressed	Weakly expressed
23	Leaf surface texture	Weakly corrugated	Weakly corrugated	Weakly corrugated	Weakly corrugated	Weakly corrugated
24	Leaf orientation	Horizontal	Horizontal	Horizontal	Horizontal	Horizontal
25	Presence of stipules	Present	Present	Present	Present	Present

26	Shape of leaf lobes	Oval	Oval	Oval	Oval	Oval
27	Dissection of lobe margins	Medium	Medium	Medium	Medium	Medium
28	Inflorescence type	Simple	Simple	Simple	Simple	Simple
29	Inflorescence structure	Compact	Compact	Compact	Compact	Compact
30	Inflorescence length	Medium (15 –25 cm)	Medium (15 – 25 cm)	Medium (15 –25 cm)	Medium (15 –25 cm)	Medium (15 –25 cm)
31	Inflorescence initiation pattern	Terminates main stem growth	Terminates main stem growth	Terminates main stem growth	Terminates main stem growth	Terminates main stem growth
32	Number of flowers per inflorescence	Medium (6 –12)	Medium (6 – 12)	Medium (6 – 12)	Medium (6 –12)	Medium (6 –12)
33	Flower size	Medium (2.3 – 3.5 cm)	Medium (2.3 – 3.5 cm)	Medium (2.3 – 3.5 cm)	Medium (2.3 – 3.5 cm)	Medium (2.3 – 3.5 cm)
34	Pedicel length	Medium (1 –2 cm)	Medium (1 –2 cm)	Medium (1 – 2 cm)	Medium (1 –2 cm)	Medium (1 –2 cm)
35	Pedicel type	Jointless (without articulation)	Jointless (without articulation)	Jointless (without articulation)	Jointless (without articulation)	Jointless (without articulation)
36;	Number of floral part	Few (5)	Few (5)	Few (5)	Few (5)	Few (5)
37	Calyx size	Smaller than the corolla	Smaller than the corolla	Smaller than the corolla	Smaller than the corolla	Smaller than the corolla
38	Sepal size	Long (>1.5 cm)	Long (>1.5 cm)	Long (>1.5 cm)	Long (>1.5 cm)	Long (>1.5 cm)
39	Degree of sepal fusion	Connate at the base	Connate at the base	Connate at the base	Connate at the base	Connate at the base
40	Sepal pubescence	Sparsely pubescent	Sparsely pubescent	Sparsely pubescent	Sparsely pubescent	Sparsely pubescent
41	Corolla coloration	Yellow-orange	Yellow-orange	Yellow-orange	Yellow-orange	Yellow-orange
42	Pistil position;	Shorter than the stamens	Shorter than the stamens	Shorter than the stamens	Shorter than the stamens	Shorter than the stamens
43	Flower type	Fertile	Fertile	Fertile	Fertile	Fertile
44	Fruit shape	Oblate	Oblate	Oblate	Oblate	Oblate
45	Fruit weight	Medium (51 – 100 g)	Medium (51 – 100 g)	Medium (51 –100 g)	Medium (51 – 100 g)	Medium (51 – 100 g)
46	Fruit surface	Moderately ribbed	Moderately ribbed	Moderately ribbed	Moderately ribbed	Moderately ribbed
47	Unripe fruit coloration	Green	Green	Green	Green	Green
48	Mature fruit coloration	Orange	Orange	Orange	Orange	Orange
49	Exocarp coloration	Colorless	Colorless	Colorless	Colorless	Colorless
50	Mesocarp coloration	Red	Red	Red	Red	Red
51	Exocarp surface	Glossy	Glossy	Glossy	Glossy	Glossy

52	Fruit base	Slightly indented	Slightly indented	Slightly indented	Slightly indented	Slightly indented
53	Fruit apex	Slight corky scar	Slight corky scar	Slight corky scar	Slight corky scar	Slight corky scar
54	Fruit locule type	Irregular	Irregular	Irregular	Irregular	Irregular
55	Number of locules per fruit	Few (5)	Few (5)	Few (5)	Few (5)	Few (5)
56	Fruit cracking	Absent	Absent	Absent	Absent	Absent
57	Number of seeds per fruit	Few (less than 50)	Few (less than 50)	Few (less than 50)	Few (less than 50)	Few (less than 50)
58	Thousand-seed weight	Medium (2 –3 g)	Medium (2 –3 g)	Medium (2 –3 g)	Medium (2 –3 g)	Medium (2 –3 g)
59	Seed coloration	Grey	Grey	Grey	Grey	Grey
60	Fruit pubescence	Weakly pubescent	Weakly pubescent	Weakly pubescent	Weakly pubescent	Weakly pubescent
61	Uniformity of fruit ripening	High	High	High	High	High

The study was conducted in accordance with the "Comprehensive Unified COMECON (Council for Mutual Economic Assistance) Descriptor List and International COMECON Descriptor List for the Genus Lycopersicon TOURN." (USSR, Leningrad, 1979, 35 pages).The Descriptor List was developed by scientists from the following institutions of the Scientific and Technical Council of COMECON Member Countries on Collections of Wild and Cultivated Plant Species: the N.I. Vavilov All-Union Research Institute of Plant Industry (Leningrad, USSR); the Institute of Plant Introduction and Genetic Resources (Sadovo, People's Republic of Bulgaria); the V. Kolarov Higher Agricultural Institute (Plovdiv, People's Republic of Bulgaria); and the Research and Breeding Institute for Vegetable Crops (Olomouc, Czechoslovak Socialist Republic).

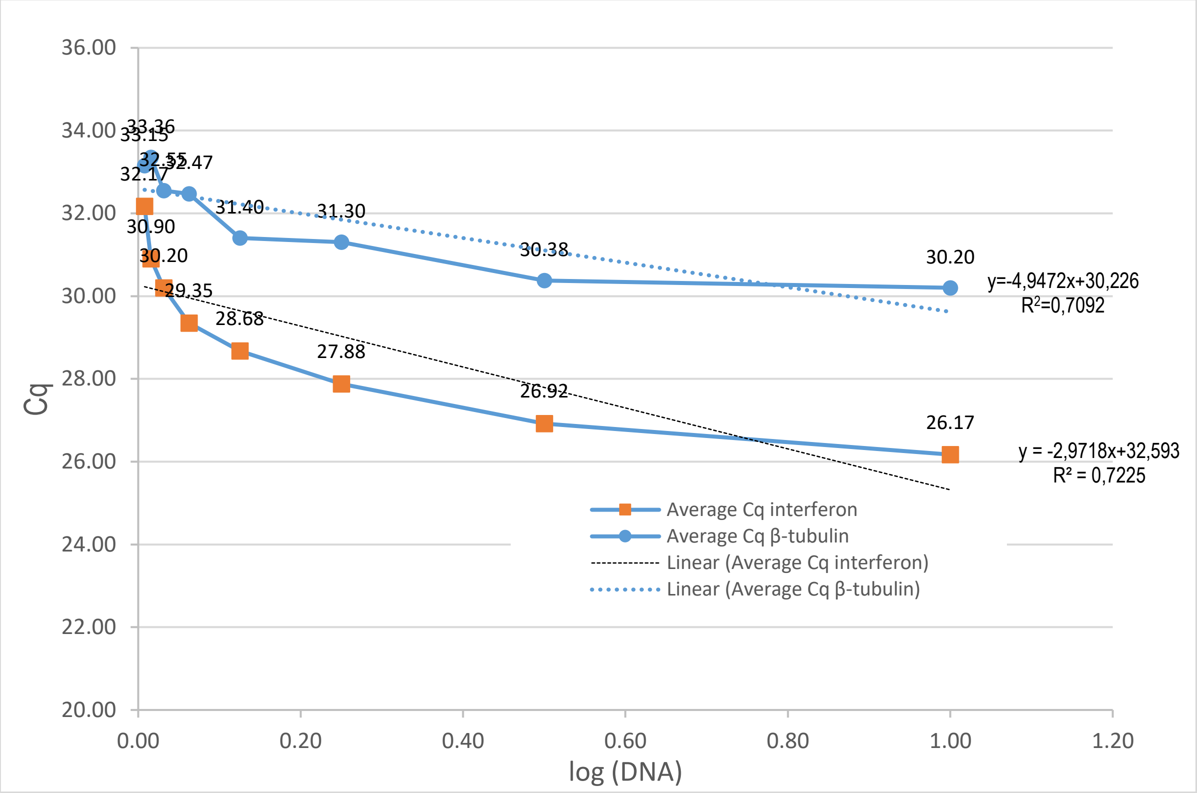

Figure S1. Standard curves of the interferon and β-tubulin qPCR assays constructed from two-fold serial dilutions of template DNA. The x-axis shows the log_2-transformed dilution factor and the y-axis the average Cq values; regression equations and coefficients of determination are shown.
